# Prevalence, causes and outcomes of hospital admissions in Mongolia: a national registry-based descriptive analysis

**DOI:** 10.1016/j.lanwpc.2025.101470

**Published:** 2025-01-20

**Authors:** Altanchimeg Sainbayar, Ganbold Lundeg, Naranpurev Mendsaikhan, Jens Meier, Martin W. Dünser

**Affiliations:** a Department of Critical Care and Anesthesia, Mongolian National University of Medical Sciences, Ulaanbaatar, Mongolia; b Intensive Care Unit, Mongolia Japan Hospital, Ulaanbaatar, Mongolia; c Department of Anesthesiology and Intensive Care Medicine, Kepler University Hospital and Johannes Kepler University, Linz, Austria

**Keywords:** Mongolia, Hospital admission, Prevalence, Admission diagnosis, Length of stay, Mortality, COVID-19

## Abstract

**Background:**

No systematic data on the prevalence of, reasons for, and outcomes of hospital admissions in Mongolia are currently available.

**Methods:**

In a descriptive analysis, the Mongolian National Hospital Data registry was screened for all hospital admissions in adults from 1 January 2019 to 31 December 2023. The endpoints were the prevalence of and most common reasons for hospital admission, the length of hospital stay, and the hospital mortality rate. Descriptive methods, Chi-square tests, Mann-Whitney-U-tests, and ANOVA on ranks were used for data analysis.

**Findings:**

3,449,083 cases were analysed. The median prevalence of hospital admissions per 100,000 population per year was 20,242 (IQR, 19,412–20,778). The most common main diagnoses were COVID-19 (8.7%; 299,409/3,449,083), chronic tubulo-interstitial nephritis (6.7%; 230,244/3,449,083), chronic arterial hypertension (2.5%; 86,213/3,449,083), chronic ischaemic heart disease (2.3%; 80,071/3,449,083), nerve root/plexus disorders (2.3%; 79,341/3,449,083), hypertensive heart disease (2.1%; 73,597/3,449,083), and lower back pain (1.8%; 60,614/3,449,083). The median length of hospital stay was 7 days (IQR, 6–9) and the hospital mortality rate was 0.66% (22,683/3,449,083). Differences in all endpoints were found between female and male participants, the periods before, during, and after the COVID-19 pandemic, regions of residence, seasons of admission, levels of admission hospitals, and public and private hospitals.

**Interpretation:**

During a five-year period including the COVID-19 pandemic, the prevalence of hospital admissions in Mongolian adults was high during the study period. An analysis of the most common diagnoses leading to hospital admission suggested that many diagnoses could be managed without hospital admission in outpatient or primary care settings.

**Funding:**

Institutional funding (10.13039/100018664Mongolian National University of Medical Sciences and the 10.13039/100031307Johannes Kepler University).

**Research in context**

**Evidence before this study**

We searched the PubMed database using the following medical subject headings terms: ((Mongolia) AND (hospital admissions) and (prevalence)). In addition, other searches with the terms ((Mongolia) AND (hospital admissions) and (causes)) as well as ((Mongolia) AND (hospital admissions) and (outomces)) were conducted. Hand searches of the reference lists of all identified publications were performed in order to find further evidence. So far, only hospital admissions for specific disease states (*e.g.*, lower respiratory tract infections) and in specific patient populations (*e.g.*, children) have been evaluated in the Mongolian population. No study has so far evaluated the overall prevalence of and main reasons for hospital admissions in Mongolia.

**Added value of this study**

This descriptive analysis of the Mongolian National Hospital Data registry reports on three-million-four-hundred-forty-nine-thousand-eighty-three adult hospital admissions over a 5 years period. The median prevalence of hospital admissions per 100,000 population per year was 20,242 (IQR, 19,412–20,778). The most common main diagnoses were COVID-19, chronic tubulo-interstitial nephritis (miscoding), chronic arterial hypertension, chronic ischaemic heart disease, nerve root/plexus disorders, hypertensive heart disease, lower back pain, cholelithiasis, lower respiratory tract infections, and other cerebrovascular diseases. The median length of hospital stay was 7 (IQR, 6–9) days and the all-cause crude hospital mortality rate 0.66%. Differences in all endpoints were found between females and males, the time periods before, during, and after the COVID-19 pandemic, regions of residence, seasons of admission, levels of admission hospitals, and between public and private hospitals.

**Implications of all the available evidence**

The prevalence of hospital admissions in Mongolian adults was very high during a five-year period including the COVID-19 pandemic. An analysis of the most common main diagnoses leading to hospital admission suggests that many of these diagnoses could be managed without hospital admission in outpatient or primary care settings, resulted in a length of stay of seven days, and a low hospital mortality. The results of this study may inform future planning and financing of hospital-based healthcare services in Mongolia.

## Introduction

Hospitals are essential components of a nation's healthcare system. Understanding the rates of, reasons for, and outcomes of hospital admissions is key for hospital capacity planning, healthcare policy development, and resource allocation to optimize healthcare services and patient outcomes.^1^ Public health emergencies, such as the recent corona virus disease 2019 (COVID-19) pandemic, do not only strain hospital services, but also have an important and lasting impact on the use of hospital resources in their aftermath.

Mongolia is a Central Asian lower-middle income country and the least densely populated country in the world.^2^ Due to substantial improvements in the healthcare system, life expectancy at birth increased from 58.8 years in 1990 to 71 years in 2021.^2^^,^^3^ Apart from geographical barriers and harsh climate conditions, the Mongolian healthcare system continues to face relevant challenges.^4^ A sub-analysis of the Global Burden of Disease study indicated that non-communicable diseases are the leading cause of premature death in Mongolia, with arterial hypertension and dietary risks being the most important risk factors.^5^ Before 1991, Mongolia implemented a Semashko-style, centralized healthcare system predominantly offering hospital-based curative health services.^6^ Accordingly, with eight hospital beds per 1000 population in 2017, Mongolia continues to have one of the highest numbers of hospital beds worldwide.^7^ The Mongolian public hospital system is organized in three tiers.^8^ At the sub-province (primary *Soum*) level, *Soum* hospitals with 15–30 beds provide both out- and inpatient services for patients with noncomplicated conditions. Province (*Aimag*) hospitals typically have 200–250 beds and serve as referral centres for sub-province hospitals and provide multispecialty care. In Ulaanbaatar, the capital city of Mongolia, district hospitals take over the role of province hospitals. Province and district hospitals make up the secondary level of the hospital-based healthcare system covering one third of Mongolia's hospital bed capacity. All tertiary-level hospitals are located in Ulaanbaatar and provide specialized services unavailable at the provincial level.^8^ During the last decades, the number of private healthcare facilities continuously increased. In 2023, 25.5% of hospital beds in Mongolia were located in private hospitals.^9^ The Mongolian healthcare system is jointly funded by the Ministry of Health budget derived from tax revenues, the mandatory Social Health Insurance Fund, and out-of-pocket payments.^10^^,^^11^ Since the Mongolian health care financing reform in 2021, the majority of healthcare costs, particularly hospital expenses, are covered by the Social Health Insurance Fund.^12^

No systematic data on the prevalence of and reasons for hospital admissions in Mongolia have so far been published. The goal of this study was to determine the prevalence of, reasons for, and outcomes of hospital admissions in Mongolian adults during the time from January 2019 until December 2023.

## Methods

This study was designed as a descriptive analysis of a national registry. It included data from all inpatient admissions to public and private hospitals in Mongolia from January 1, 2019 until December 31, 2023. The study protocol was evaluated and approved by the Ethics Committee of the Mongolian National University of Medical Sciences (2021/3–11). In view of the retrospective study design written informed consent was waived. The manuscript was prepared according to the updated STROBE checklist for reporting cohort studies.^13^

### The National Hospital Data registry

The National Hospital Data registry is hosted by the National Statistics Office of Mongolia. This registry aggregates data on all inpatient admissions to public and private hospitals in Mongolia. Using a standardized online data entry tool, patient-related, medical and administrative data of each hospital admission are entered by a dedicated physician trained in the use of the online data collection tool. Variables and data entry into the online registry are standardized and summarized in a special guidebook. All data are aggregated, updated daily, and monitored at the Centre for Health Development of Mongolia. The latter institution assures completeness of the datasets by retrieving missing data from submitting bodies at regular intervals. Following data clearance by the Mongolian Centre for Health Development, data are then transferred to the National Statistics Office of Mongolia.^12^

### Study population and definitions

All inpatient admissions to a hospital during the study period which had been recorded in the National Hospital Data registry were eligible for study inclusion. Records without patient data, with missing demographic patient data (defined as either age or sex), as well as records of patients <18 years were excluded. A hospital was defined as a healthcare institution which (1) offers diagnostic and therapeutic patient services for medical conditions, (2) has at least six beds, (3) has an organized physician staff, and (4) provides continuous nursing services under the supervision of a registered nurse.^14^ An admission was defined as a course of inpatient treatment in a hospital with an intended duration of at least one overnight stay.^15^ For the purpose of this study, hospital admissions were counted per admission and not per patient meaning that individual patients with repeated hospital admissions or those who were transferred from one hospital to another were included into the analysis more than once.

### Study variables

The following data were extracted from the National Hospital Data registry for all study participants: sex, age, region of residence, date of hospital admission, level of admission hospital, main diagnosis leading to hospital admission as indicated by the International Statistical Classification of Diseases and Related Health Problems (ICD)-10 code,^16^ length of hospital stay, and survival status at hospital discharge.

To calculate the prevalence of hospital admissions per 100,000 population per year, the number of registered inhabitants per province and region in each of the five study years was collected from the National Statistics Office of Mongolia. The regions of residence were grouped into the following six regions of Mongolia (Electronic Supplementary Material Figure E1): East: Dornod, Sukhbataar, and Khentii province; North: Arkhangai, Bulgan, Orkhon, Khuvsgul, Selenge, and Darkhna-Uul province; Centre: Dornogobi, Tuv and Uvurkhangai province; South: Bayankhongor, Umnugobi, Gobisumber, and Dundgobi province; West: Bayan-Ulgii, Gobi-Altai, Zavkhan, Uvs, and Khovd province; Ulaanbaatar: capital city of Ulaanbaatar.^17^ Based on the date of hospital admission, the season of admission was determined (winter: December 1—February 28, mean temperature: −17.4 °C; spring: March 1—May 31, mean temperature: 2.7 °C; summer: June 1—August 31, mean temperature: 17.6 °C; autumn: September 1—November 30, mean temperature: 1.1 °C).^18^ Using COVID-19 case records of Mongolia as published by the World Health Organization,^19^ the five study years were categorized into a time period before (January 1, 2019 until December 31, 2020), during (January 1, 2021 until December 31, 2022), and after the COVID-19 pandemic (January 1 until December 31, 2023). Admission hospitals were grouped into level 1 (*Soum* hospitals), level 2 (provincial hospitals, district hospitals, private hospitals), and level 3 (university hospitals, specialized hospitals), as well as into public and private hospitals. In order to identify the diagnostic category and admission diagnosis leading to hospital admission, the ICD-10 code was truncated to blocks by chapters (diagnostic categories) and to the first decimal (main diagnoses). The survival status at hospital discharge was coded as alive or dead. The all-cause crude mortality rate was then calculated as the ratio between the number of patients who died during their hospital admission and the total number of hospital admissions.

### Data processing

Study variables were extracted from the registry for each year and then merged into one datafile. Plausibility of the dataset was tested by tabling all study variables in order to identify relevant outliers. In addition, data type checks were performed, assuring that non-numeric and numeric variables were not mixed. Wherever possible, data corrections were made. Otherwise, variables were marked as missing. No data imputation methods were used in case of missing values. Non-numeric variables of the dataset (*e.g.*, region of residence) were translated into English using an online available multilingual neural machine translated service (Google Translate®; Google, Mountain View, United States). Using the ICD package for R, ICD-10 codes were transformed into diagnostic categories and main diagnoses.

### Study endpoints

The primary study endpoint was the prevalence of hospital admissions per 100,000 population per year. Secondary study endpoints were the most common reasons for hospital admission as expressed by the ICD-10-defined diagnostic categories and the ICD-10 coded main diagnoses, the length of hospital stay, as well as the all-cause crude mortality rate at hospital discharge. Primary and secondary study endpoints were compared between males and females, the time periods before, during, and after the COVID-19 pandemic, regions of residence, seasons of admission, levels of admission hospitals, as well as between public and private hospitals.

### Statistical analysis

All statistical analyses were performed using the R software package (R version 4.2.3; The R Foundation, Vienna, Austria). Descriptive methods were used to report primary and secondary study endpoints. Kolmogorov–Smirnov tests were applied to test for normality distribution of continuous variables. As normality assumption was not fulfilled for most of these variables, comparisons of continuous variables between females and males, the time periods before, during, and after the COVID-19 pandemic, regions of residence, seasons of admission, levels of hospital admissions, as well as between public and private hospitals were conducted using Mann-Whitney-U-tests or analyses of variance (ANOVA) on ranks, as appropriate. Continuous variables are presented as median values with interquartile ranges. Categorical variables are presented as absolute numbers with percentages and were compared between subgroups using Chi-square tests (both for 2 × 2 contingency tables and contingency tables with multiple rows and/or categories). Two-sided *p*-values <0.001 were considered to indicate statistical significance, since then even after application of Bonferroni corrections for multiple comparisons between subgroups an overall significance level of 0.05 was guaranteed.

### Role of the funding source

This study was supported by institutional funds only. The Mongolian National University of Medical Sciences and the Johannes Kepler University had no influence on the study design, data collection, data analysis, interpretation of the results, writing of the manuscript, and in the decision to submit the paper for publication.

## Results

Out of 4,378,364 records in the National Hospital Data registry, 3,449,083 cases were included into the statistical analysis (Electronic Supplementary Material Figure E2). Zero-point-four percent of all data were missing (Electronic Supplementary Material Figure E3). Demographic data, regions of residence, seasons of admission, as well as level and type of admission hospital in all patients, and those admitted before, during, and after the COVID-19 pandemic are shown in Table 1.

**Table 1:** Demographic data, region of residence, season of admission, as well as level and type of admission hospital in all patients, and those admitted before, during, and after the COVID-19 pandemic.

		All	Before COVID-19 (2019–2020)	During COVID-19 (2021–2022)	After COVID-19 (2023)
	*n*	3,449,083	1,263,571	1,435,215	750,297
**Female sex**	*n* (%)	2,171,360 (63%)	794,081 (62.8%)	902,332 (62.9%)	474,947 (63.3%)
**Male sex**	*n* (%)	1,277,723 (37%)	469,490 (37.2%)	532,883 (37.1%)	275,350 (36.7%)
**Age**	years	50.1 (36.1–62.1)	49.4 (35.6–61.4)	49.5 (35.7–61.9)	52.2 (38.1–63.7)
**Region of residence**^a^	*n* (%)				
East		239,201 (6.9%)	88,252 (7%)	101,281 (7.1%)	49,668 (6.6%)
North		685,559 (19.9%)	250,739 (19.8%)	290,932 (20.3%)	143,888 (19.2%)
Centre		290,702 (8.4%)	110,410 (8.7%)	123,578 (8.6%)	56,714 (7.6%)
South		244,894 (7.1%)	90,389 (7.2%)	106,328 (7.4%)	48,177 (6.4%)
West		397,877 (11.5%)	149,067 (11.8%)	166,704 (11.6%)	82,106 (10.9%)
Ulaanbaatar		1,468,491 (42.6%)	548,253 (43.4%)	612,600 (42.7%)	307,638 (41%)
Missing		122,359 (3.3%)	26,461 (2.1%)	33,792 (2.4%)	62,106 (8.3%)
**Season of admission**	*n* (%)				
Winter		805,456 (23.4%)	305,532 (24.2%)	427,330 (29.8%)	196,695 (26.2%)
Spring		886,535 (25.7%)	346,248 (27.4%)	338,807 (23.6%)	201,480 (26.9%)
Summer		827,535 (24%)	303,869 (24%)	348,131 (24.3%)	175,535 (23.4%)
Autumn		929,557 (27%)	307,922 (24.4%)	320,947 (22.4%)	176,587 (23.5%)
**Level of admission hospital**	*n* (%)				
Level I		572,301 (16.6%)	213,947 (16.9%)	236,607 (16.5%)	121,747 (16.2%)
Level II		2,302,244 (66.8%)	823,432 (65.2%)	981,762 (68.4%)	497,050 (66.2%)
Level III		574,538 (16.7%)	226,192 (17.9%)	216,846 (15.1%)	131,500 (17.5%)
**Type of admission hospital**	*n* (%)				
Public		2,563,091 (74.3%)	965,246 (76.4%)	1,049,182 (73.1%)	548,663 (73.1%)
Private		885,944 (25.7%)	298,325 (23.6%)	386,033 (26.9%)	201,586 (26.9%)
Missing		48 (0.001%)	0 (0%)	0 (0%)	48 (0.006%)

COVID-19, corona virus disease 2019.

a Regions include the following provinces: East: Dornod, Sukhbataar, and Khentii; North: Arkhangai, Bulgan, Orkhon, Khuvsgul, Selenge, and Darkhna-Uul; Centre: Dornogobi, Tuv and Uvurkhangai; South: Bayankhongor, Umnugobi, Gobisumber, and Dundgobi, West: Bayan-Ulgii, Gobi-Altai, Zavkhan, Uvs, and Khovd; Ulaanbaatar: capital city of Ulaanbaatar.

### Prevalence of hospital admissions per 100,000 population per year

**Fig. 1:**
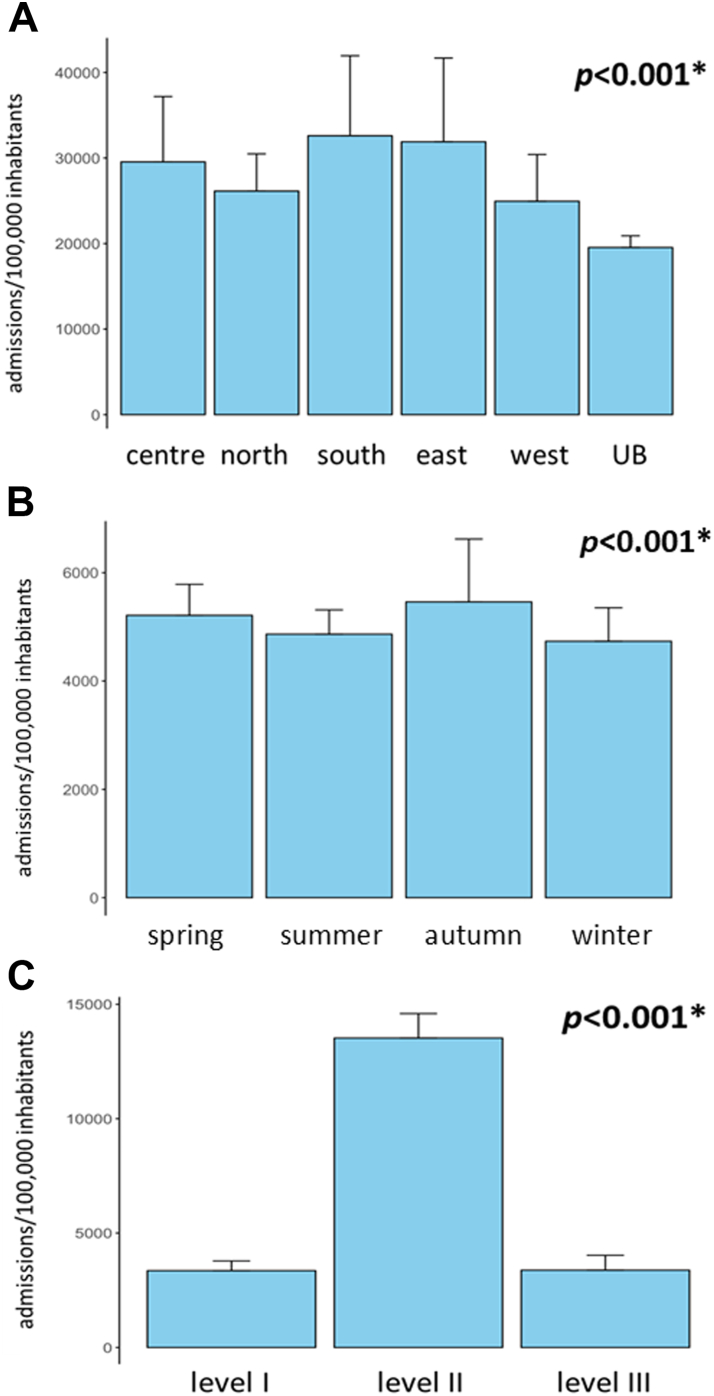
Prevalence of hospital admissions per 100,000 population for regions of residence (A), seasons of admission (B), and levels of admission hospitals (C). UB, Ulaanbaatar. ∗, significant difference between subgroups (ANOVA on ranks).

The median prevalence of hospital admissions per 100,000 population per year was 20,242 (IQR, 19,412–20,778). Electronic Supplementary Material Figure E4 displays the absolute number of hospital admissions and age distribution among male and female study patients. The median prevalence of hospital admissions per 100,000 population per year significantly differed between females and males [13,131 (12,384–13,759) vs. 7525 (7444–7910); *p* < 0.0001, Mann-Whitney-U-test], the time periods before, during, and after the COVID-19 pandemic (18,988 vs. 20,889 vs. 21,553), public and private hospitals [15,709 (14,852–15,762) vs. 5436 (4986–5792); *p* < 0.0001, Mann-Whitney-U-test], as well as between the regions of residence, seasons of admission, and levels of admission hospitals (Fig. 1).

### Diagnostic categories and main diagnoses

**Fig. 2:**
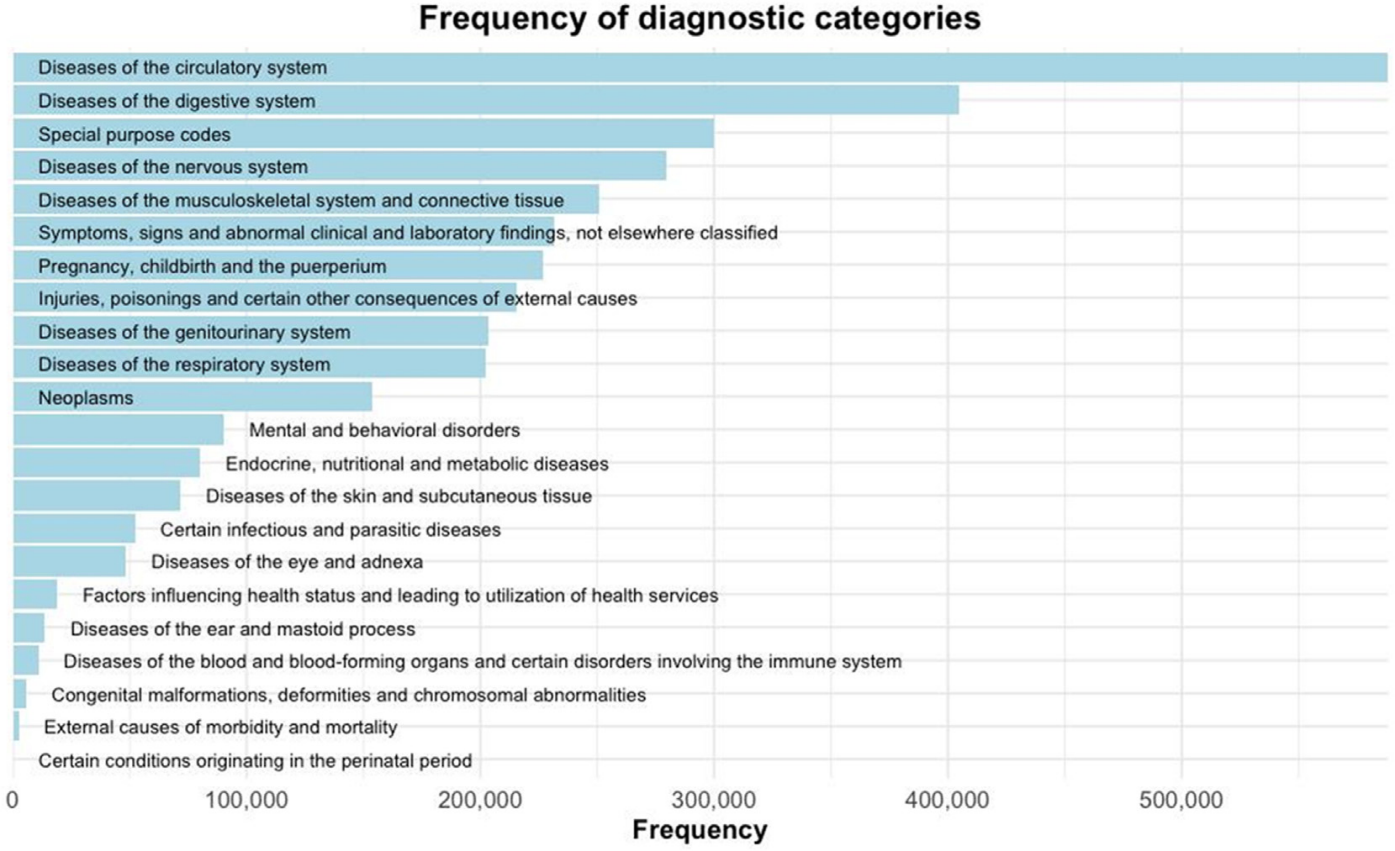
Frequency of diagnostic categories leading to hospital admission in the study population.

Diseases of the circulatory system, diseases of the digestive system, and special purpose codes were the three most frequent diagnostic categories leading to hospital admission (Fig. 2). COVID-19, chronic tubulo-interstitial nephritis, and chronic arterial hypertension were the most frequent main diagnoses leading to hospital admission in this population (Table 2). The twenty most common main diagnoses stratified by different subgroups are presented in Tables 2 and 3, as well as Electronic Supplementary Tables E1–E4. The main diagnoses leading to hospital admission significantly differed between females and males (*p* < 0.0001, Chi-square test), the time periods before, during, and after the COVID-19 pandemic (*p* < 0.0001, Chi-square test), regions of residence (*p* < 0.0001, Chi-square test), seasons of admission (*p* < 0.0001, Chi-square test), levels of admission hospitals (*p* < 0.0001, Chi-square test), as well as between public and private hospitals (*p* < 0.0001, Chi-square test).

**Table 2:** The twenty most common main diagnoses in all patients as well as in males and females.

All			Females			Males		
*n* = 3,449,083			*n* = 2,171,360			*n* = 1,277,723		
1	COVID-19	299,409 (8.7%)	1	COVID-19	189,964 (8.7%)	1	COVID-19	109,445 (8.6%)
2	Chronic tubulo-interstitial nephritis	230,244 (6.7%)	2	Chronic tubulo-interstitial nephritis	171,100 (7.9%)	2	Chronic tubulo-interstitial nephritis	59,144 (4.6%)
3	Chronic arterial hypertension	86,213 (2.5%)	3	Chronic arterial hypertension	54,168 (2.5%)	3	Chronic ischaemic heart disease	35,543 (2.8%)
4	Chronic ischaemic heart disease	80,071 (2.3%)	4	Nerve root and plexus disorders	49,854 (2.3%)	4	Chronic arterial hypertension	32,045 (2.5%)
5	Nerve root/plexus disorders	79,341 (2.3%)	5	Hypertensive heart disease	48,152 (2.2%)	5	Nerve root and plexus disorders	29,487 (2.3%)
6	Hypertensive heart disease	73,597 (2.1%)	6	Chronic ischaemic heart disease	44,528 (2.1%)	6	Alcohol-related disorders	27,667 (2.2%)
7	Lower back pain	60,614 (1.8%)	7	Complications of pregnancy termination	40,565 (1.9%)	7	Hypertensive heart disease	25,445 (2%)
8	Cholelithiasis	54,293 (1.6%)	8	False/premature labour	40,233 (1.9%)	8	Diabetes mellitus type II	24,180 (1.9%)
9	Lower respiratory tract infections	53,332 (1.5%)	9	Cholelithiasis	37,936 (1.7%)	9	Sequelae of cerebrovascular disease	23,794 (1.9%)
10	Other cerebrovascular diseases	51,944 (1.5%)	10	Other cerebrovascular diseases	37,561 (1.7%)	10	Lower back pain	23,497 (1.8%)
11	Diabetes mellitus type II	51,659 (1.5%)	11	Lower back pain	37,117 (1.7%)	11	Lower respiratory tract infections	22,579 (1.8%)
12	Liver fibrosis or cirrhosis	49,075 (1.4%)	12	Lower respiratory tract infections	30,753 (1.4%)	12	Liver fibrosis or cirrhosis	21,442 (1.7%)
13	Other headache syndromes	42,777 (1.2%)	13	Other headache syndromes	29,948 (1.4%)	13	Liver and bile duct malignancy	19,946 (1.6%)
14	Sequelae of cerebrovascular disease	42,656 (1.2%)	14	Liver fibrosis or cirrhosis	27,633 (1.3%)	14	Angina pectoris	18,071 (1.4%)
15	Angina pectoris	41,023 (1.2%)	15	Diabetes mellitus type II	27,479 (1.3%)	15	Acute appendicitis	17,641 (1.4%)
16	Complications of pregnancy termination	40,565 (1.2%)	16	Knee osteoarthritis	24,825 (1.1%)	16	Cholelithiasis	16,357 (1.3%)
17	False/premature labour	40,233 (1.2%)	17	Angina pectoris	22,952 (1.1%)	17	Chronic obstructive pulmonary disease	15,913 (1.2%)
18	Liver and bile duct malignancy	37,378 (1.1%)	18	Other abnormal products of conception	22,664 (1%)	18	Intracranial injury	14,822 (1.2%)
19	Acute appendicitis	36,086 (1%)	19	Gastritis and duodenitis	20,982 (1%)	19	Chronic hepatitis	14,783 (1.2%)
20	Gastritis and duodenitis	34,297 (1%)	20	Polyosteoarthritis	20,400 (0.9%)	20	Malignancy of the stomach	14,413 (1.1%)

COVID-19, corona virus disease 2019.

**Table 3:** The twenty most common main diagnoses in all patients before, during and after the COVID-19 pandemic.

Before COVID-19 (2019–2020)			During COVID-19 (2021–2022)			After COVID-19 (2023)		
*n* = 1,263,571			*n* = 1,435,215			*n* = 750,297		
1	Chronic tubulo-interstitial nephritis	112,147 (8.9%)	1	COVID-19	297,996 (20.8%)	1	Chronic tubulo-interstitial nephritis	33,165 (4.4%)
2	Chronic arterial hypertension	65,837 (5.2%)	2	Chronic tubulo-interstitial nephritis	84,932 (5.9%)	2	Hypertensive heart disease	23,712 (3.2%)
3	Nerve root/plexus disorders	46,019 (3.6%)	3	Chronic ischaemic heart disease	31,182 (2.2%)	3	Diabetes mellitus type II	13,384 (1.8%)
4	Chronic ischaemic heart disease	35,646 (2.8%)	4	Hypertensive heart disease	28,468 (2%)	4	Chronic ischaemic heart disease	13,243 (1.8%)
5	Lower back pain	25,742 (2%)	5	Lower back pain	24,337 (1.7%)	5	Cholelithiasis	12,202 (1.6%)
6	Lower respiratory tract infections	22,850 (1.8%)	6	Cholelithiasis	21,819 (1.5%)	6	Nerve root/plexus disorders	11,989 (1.6%)
7	Complications of pregnancy termination	22,132 (1.8%)	7	Nerve root/plexus disorders	21,333 (1.5%)	7	Other cerebrovascular diseases	11,927 (1.6%)
8	Hypertensive heart disease	21,417 (1.7%)	8	Diabetes mellitus type II	19,875 (1.4%)	8	Lower respiratory tract infections	11,231 (1.5%)
9	Other headache syndromes	20,871 (1.7%)	9	Other cerebrovascular diseases	19,678 (1.4%)	9	Liver fibrosis or cirrhosis	10,700 (1.4%)
10	Other cerebrovascular diseases	20,339 (1.6%)	10	Lower respiratory tract infections	19,251 (1.3%)	10	Acute nephritis	10,571 (1.4%)
11	Cholelithiasis	20,272 (1.6%)	11	Liver fibrosis or cirrhosis	18,961 (1.3%)	11	Lower back pain	10,535 (1.4%)
12	Angina pectoris	20,172 (1.6%)	12	Angina pectoris	17,005 (1.2%)	12	Other arthritis	10,101 (1.3%)
13	Sequelae of cerebrovascular disease	19,523 (1.5%)	13	Chronic arterial hypertension	16,067 (1.1%)	13	Sequelae of cerebrovascular disease	9940 (1.3%)
14	Gastritis and duodenitis	19,440 (1.5%)	14	Acute appendicitis	14,323 (1%)	14	Liver and bile duct malignancy	9490 (1.3%)
15	Liver fibrosis or cirrhosis	19,414 (1.5%)	15	Liver and bile duct malignancy	13,870 (1%)	15	Heart failure	8805 (1.2%)
16	Diabetes mellitus type II	18,400 (1.5%)	16	Complications of pregnancy termination	13,674 (1%)	16	Other headache syndromes	8504 (1.1%)
17	False/premature labour	18,197 (1.4%)	17	False/premature labour	13,617 (0.9%)	17	Alcohol-related disorders	8447 (1.1%)
18	Chronic hepatitis	17,942 (1.4%)	18	Other headache syndromes	13,402 (0.9%)	18	False/premature labour	8419 (1.1%)
19	Acute appendicitis	15,704 (1.2%)	19	Sequelae of cerebrovascular disease	13,193 (0.9%)	19	Cervical spondylosis	7248 (1%)
20	Other diseases of the pancreas	15,637 (1.2%)	20	Alcohol-related disorders	11,849 (0.8%)	20	Knee osteoarthritis	7167 (1%)

COVID-19, corona virus disease 2019.

### Length of hospital stay

The median length of hospital stay in the study population was 7 (IQR, 6–9) days (Electronic Supplementary Material Figure E5). The median length of stay in the hospital significantly differed between females and males, the time period before, during, and after the COVID-19 pandemic, regions of residence, seasons of admission, levels of admission hospitals, as well as between public and private hospitals (Electronic Supplementary Material Table E5).

### All-cause crude hospital mortality rate

The all-cause crude hospital mortality rate in the study population was 0.66% (22,683/3,449,083). The hospital mortality rate significantly differed between females and males, the time period before, during, and after the COVID-19 pandemic, regions of residence, seasons of admission, levels of admission hospitals, as well as between public and private hospitals (Electronic Supplementary Material Table E6). The all-cause crude hospital mortality rates of the twenty most common main diagnoses ranged between 0.002% (1/42,777; other headache syndromes) and 5.7% (2115/37,378; liver and bile duct malignancy) (Table 4). Liver and bile duct malignancy, COVID-19, and nontraumatic intracerebral haemorrhage were the main diagnoses causing most hospital deaths, whereas sepsis, vascular disorders of the intestines, and respiratory failure were the main diagnoses with the highest crude hospital mortality rates (Table 4).

**Table 4:** Crude hospital mortality rates of the twenty most common main diagnoses, the twenty main diagnoses causing most hospital deaths, and the twenty main diagnoses with the highest crude hospital mortality rate.

20 most common admission diagnoses and associated crude hospital mortality			20 admission diagnoses causing most hospital deaths			20 admission diagnoses with the highest crude hospital mortality		
		Mortality			Death cases			Mortality
		*n* (%)			*n* (% of all deaths)			*n* (%)
1	COVID-19	2036 (0.7%)	1	Liver and bile duct malignancy	2115 (9.3%)	1	Sepsis other than streptococcal or puerperal	210 (35.7%)
2	Chronic tubulo-interstitial nephritis	28 (0.01%)	2	COVID-19	2036 (9%)	2	Vascular disorders of the intestines	42 (34.7%)
3	Chronic arterial hypertension	41 (0.05%)	3	Nontraumatic intracerebral haemorrhage	2020 (8.9%)	3	Respiratory failure, unspecified	162 (22%)
4	Chronic ischaemic heart disease	410 (0.5%)	4	Liver fibrosis or cirrhosis	1326 (5.8%)	4	Alzheimer disease	26 (20.2%)
5	Nerve root/plexus disorders	0 (0%)	5	Lower respiratory tract infections	1111 (4.9%)	5	Nontraumatic intracerebral haemorrhage	2020 (14.8%)
6	Hypertensive heart disease	40 (0.05%)	6	STEMI & NSTEMI	693 (3.1%)	6	Lymphoid leukaemia	48 (14.3%)
7	Lower back pain	3 (0.005%)	7	Malignancy of the stomach	683 (3%)	7	Miliary tuberculosis	241 (14%)
8	Cholelithiasis	62 (0.1%)	8	Cerebral infarction	525 (2.3%)	8	Diverticular disease of the intestines	25 (12.8%)
9	Lower respiratory tract infections	1111 (2.1%)	9	Malignancy of the bronchus & lung	442 (1.9%)	9	Non-Hodgkin lymphoma	16 (11.7%)
10	Other cerebrovascular diseases	24 (0.05%)	10	Chronic ischaemic heart disease	410 (1.8%)	10	Aortic aneurysm/dissection	38 (11.6%)
11	Diabetes mellitus type II	410 (0.8%)	11	Diabetes mellitus type II	410 (1.8%)	11	Myeloid leukaemia	103 (11%)
12	Liver fibrosis or cirrhosis	1326 (2.7%)	12	Nontraumatic subarachnoid haemorrhage	375 (1.7%)	12	Nontraumatic subarachnoid haemorrhage	375 (10.8%)
13	Other headache syndromes	1 (0.002%)	13	Cardiomyopathy	370 (1.6%)	13	Mature T/NK-cell lymphoma	19 (10%)
14	Sequelae of cerebrovascular disease	164 (0.4%)	14	Respiratory tuberculosis	329 (1.5%)	14	Maternal infection	45 (9.5%)
15	Angina pectoris	37 (0.1%)	15	Chronic kidney disease	258 (1.1%)	15	Abscess of the lung or mediastinum	71 (8.5%)
16	Complications of pregnancy termination	1 (0.002%)	16	Acute pancreatitis	248 (1.1%)	16	Liver failure	42 (8.3%)
17	False/premature labour	0 (0%)	17	Miliary tuberculosis	241 (1.1%)	17	STEMI & NSTEMI	693 (7.7%)
18	Liver and bile duct malignancy	2115 (5.7%)	18	Malignancy of the oesophagus	232 (1%)	18	Other diseases of the digestive tract	70 (7.2%)
19	Acute appendicitis	11 (0.03%)	19	Malignancy of the pancreas	226 (1%)	19	Bacterial meningitis	18 (7.2%)
20	Gastritis and duodenitis	22 (0.1%)	20	Chronic obstructive pulmonary disease	199 (0.9%)	20	Malignancy of the brain	60 (7.1%)

COVID-19, corona virus disease 2019; NSTEMI, non-ST-elevation myocardial infarction; STEMI, ST-elevation myocardial infarction.

## Discussion

In this descriptive analysis of the Mongolian National Hospital Data registry, a median prevalence of 20,242 adult hospital admissions per 100,000 population per year was observed. The most common main diagnoses were COVID-19 followed by chronic tubulo-interstitial nephritis, as well as other low acuity or chronic conditions such as chronic arterial hypertension, chronic ischaemic heart disease, nerve root/plexus disorders, hypertensive heart disease, and lower back pain. The median length of hospital stay was seven days, and the all-cause crude hospital mortality 0.66% (22,683/3,449,083). Differences in all study endpoints were found between females and males, the time periods before, during, and after the COVID-19 pandemic, regions of residence, seasons of admission, levels of admission hospitals, as well as between public and private hospitals.

This is the first systematic and nationwide analysis of hospital admissions in Mongolian adults. Strengths of our analysis are the high case number analysed and the very low rate of missing data. The study period spanned over the periods before (2019–2020), during (2021–2022), and after (2023) the COVID-19 pandemic in Mongolia, allowing to assess the impact of the pandemic on hospital admissions in the country. All data were extracted from the National Hospital Data registry which contains clearly defined variables, is systematically monitored, and well maintained as reflected by the extremely low rate of cases that had to be excluded because of missing demographic data.

The key finding of our study was the prevalence of 20,242 (IQR, 19,412–20,778) adult hospital admissions per 100,000 population per year. In comparison with other countries, this prevalence is exceptionally high. In 2021, the average number of hospital discharges per 100,000 population in OECD countries was 13,050.^20^ Only two countries in the OECD group, Germany and Austria, had higher annual hospital discharge rates per 100,000 population than Mongolia.^20^ Other middle-income countries, such as Mexico or Costa Rica, reported hospital discharge rates per 100,000 population of 3010 and 4630 in 2021, respectively. In 2021, neighbouring China counted 17,280 hospital discharges per 100,000 population.^20^ When comparing these data, it is important to remember that the results of our study only relate to adult patients while the data published for the OECD countries summarize both paediatric and adult hospital admissions.

The very high prevalence of hospital admissions among Mongolian adults likely reflects the legacy of the healthcare system implemented during socialism which almost exclusively depended on hospital-based services and fostered lengthy hospital stays also for low acuity conditions.^21^ A previous study from Mongolia indicated that inefficient referral pathways might be another reason for the high prevalence of hospital admissions in Mongolia.^22^ In the latter study, inappropriate self-referral pathways which bypass official healthcare structures such as primary health centres were common and resulted in high costs caused by unnecessary hospital stays.^22^ Other factors may have contributed to high hospitalization rates, as well. Among these are healthcare reimbursement practices favouring in-over outpatient care, urban-rural disparities in outpatient healthcare facilities, as well as cultural and community beliefs preferring hospital admission for both acute and chronic health conditions.

Overall, the high rate of hospital admissions, particularly of patients with low-acuity (*e.g.*, lower back pain) or chronic conditions (*e.g.*, chronic arterial hypertension, chronic ischaemic heart disease, diabetes mellitus) can be interpreted as an overuse of hospital resources, which could be used for patients with more complex diseases instead. This interpretation is supported by scientific data that several of these conditions, also referred to as “ambulatory care sensitive conditions”, can safely and effectively be managed in primary care settings.^23^, ^24^, ^25^ Outside of the COVID-19 pandemic, chronic tubulo-interstitial nephritis was surprisingly found to be the most common main diagnosis in this study population. According to the health officials of the Mongolian Centre for Health Development and a separate data analysis, this ICD-10 code (N11) was used as a summary code for chronic inflammatory kidney diseases (*e.g.*, chronic tubulo-interstitial nephritis, chronic pyelonephritis). Another reason why this ICD-10 code was over-represented in this population could be that there is widespread belief among Mongolian physicians that common unspecific symptoms (*e.g.*, chronic fatigue) are related to chronic inflammatory diseases of the kidneys. As indicated in Table 2, the latter is a frequent cause why Mongolian adults, particularly females, seek inpatient hospital care. An effort was made by insurance companies to reduce reimbursement sums for this diagnostic code from 2023 on. This could explain why there was a decrease in the frequency of this diagnosis in 2023, however, paralleled by an increase in the frequency of acute nephritis.

The prevalence of hospital admissions per 100,000 population per year increased during the COVID-19 pandemic years in Mongolia (2021–2022). When deducting the number of hospital admissions due to COVID-19 from the total number of hospital admissions during this time period, there was an approximately 10% reduction in non-COVID-19 admissions compared to the time period before COVID-19 in Mongolia (2019–2020). This decrease was less pronounced than reported from other countries.^26^^,^^27^ The finding that hospital mortality rates during the COVID-19 pandemic years in Mongolia were only slightly higher than those during the years before and after the COVID-19 pandemic [0.73% (10,517/1,435,215) vs. 0.59% (7453/1,263,571) and 0.63% (4713/750,297), respectively] mirrors the effective COVID-19 pandemic response in Mongolia.^28^^,^^29^ Interestingly, the number of hospital admissions continued to rise in 2023, despite a dramatic drop in COVID-19 cases. In 2023, once again and with minor modifications compared to the time before COVID-19, low-acuity and chronic conditions were the most common main diagnoses leading to hospital admission.

Despite a median male to female ratio in the Mongolian population of 0.97 during the study period,^30^ the prevalence of hospital admissions was double as high among females than males. Although certain main diagnoses were more common among or exclusive to females (*e.g.*, obstetric/gynaecological diagnoses), these differences can only partly explain the higher rate of hospital admissions in females. Of all Mongolian regions, Ulaanbaatar had the lowest prevalence of hospital admissions in the country. A steadily increasing number of primary healthcare centres in the capital city could be one explanation for this observation.^21^ Hospital admissions were particularly prevalent during spring and autumn compared to winter and summer months. Higher workloads during winter months as well as climate conditions together with poor road infrastructure in Mongolia making journeys to hospitals both lengthy and risky during winter might partly explain this finding.^31^ Corresponding to the fact that secondary level hospitals account for almost two thirds of the total hospital bed capacity in Mongolia, the prevalence of admissions to these hospitals were higher than to primary and tertiary level hospitals.

The analysis of the most common main diagnoses by the level of admission hospital highlights further important findings of this study. First, chronic and low-acuity conditions also range among the most common main diagnoses in tertiary level hospitals. Although the practice of admitting patients to hospitals because of such diagnoses needs to be critically reviewed across all hospital levels, admitting those patients to tertiary level hospitals appears to particularly strain valuable hospital resources required for patients with severe and/or complex diseases. Another key result is the fact that false/premature labour was the eleventh most common main diagnosis in secondary level hospitals and outnumbered hospital admissions for uncomplicated childbirth by far (Fig. 2). This observation reflects the governmental regulation that primary level hospitals in Mongolia do not offer maternity services, not even for uncomplicated pregnancies, but early referral of pregnant women to secondary or tertiary level hospitals is encouraged.

Hospital length of stay is an important indicator of effective planning and management of hospital bed resources. The median length of stay in the hospital in this population and across all subgroups was seven days. Although a median length of stay of one week is in line with reports from other countries, where the average length of hospital stay similarly ranged between five and seven days,^32^ the histogram of our data highlights that over one third of hospitalized adults in Mongolia are discharged on hospital day seven. A key explanation for this striking finding is the hospital reimbursement policy in Mongolia according to which hospital expenses are only refunded if an inpatient treatment exceeds five days. As a consequence, both doctors, patients and families have become accustomed to prolonged hospital stays, even for low acuity or chronic conditions.

The all-cause crude hospital mortality rate in this population was low [0.66% (22,683/3,449,083)]. The high rate of main diagnoses, which could typically be managed on an outpatient basis, appears to be an important reason for this finding. Another remarkable result was that the hospital mortality rate of males was almost three times higher than the one of females. Relevantly higher hospital mortality rates were also observed in tertiary level hospitals likely reflecting the higher disease severity and complexity of patients admitted to these institutions. Whereas the twenty most common main diagnoses were associated with very low mortality rates, the majority of hospital deaths were attributable to disease conditions known to range among the most common causes of death in Mongolia.^5^ Apart from COVID-19, these were liver and bile duct malignancies, nontraumatic intracerebral haemorrhage, liver fibrosis and cirrhosis, lower respiratory tract infections, and myocardial infarction. Another relevant finding of our study was that sepsis was the single admission diagnosis with the highest diagnosis-related hospital mortality.

Important limitations need to be considered when interpreting the results of our study. First, only a relatively limited number of variables were analysed. This did not allow for a more detailed analysis of hospital therapies and specific causes of death. Second, despite of clear definitions and a very low rate of missing data in the national registry, the registry data have not been systematically validated. This is particularly relevant for the reliable interpretation of diagnostic categories and main diagnoses leading to hospital admissions. The obvious miscoding of chronic tubulo-interstitial nephritis appears to be a prominent example of such a systematic coding mistake. Although we found no other indications in our study results, we cannot exclude that further systematic errors have occurred and that the common reasons for hospital admission as expressed by the ICD-10-defined diagnostic categories and the ICD-10 coded main diagnoses in this study reflect the true admission diagnoses in this population. In order to ensure future data validation, systematic data quality checks should be introduced into the National Hospital Data registry. Third, all comparisons between subgroups yielded *p*-values <0.0001 indicating statistical significance even after application of strict Bonferroni correction rules. This is likely the consequence of the high case numbers analysed and might not always indicate relevant differences between subgroups. For example, minor differences in the hospital length of stay between subgroups might have been statistically significant but this difference does not appear to be clinically relevant. Finally, we do not report ethnicity data.

In conclusion, the prevalence of hospital admissions in Mongolian adults was very high during a five-year period including the COVID-19 pandemic. An analysis of the most common main diagnoses leading to hospital admission suggests that many of these diagnoses could be managed without hospital admission in outpatient or primary care settings, resulted in a length of stay of seven days and a low hospital mortality. The results of this study may inform future planning and financing of hospital-based healthcare services in Mongolia.

## Contributors

AS designed the study, directly accessed and verified the data, collected data, interpreted results, and drafted the manuscript. GL designed the study, directly accessed and verified the data, collected data, interpreted the results, and revised the manuscript for important intellectual content. NM designed the study, directly accessed and verified the data, collected data, interpreted the results, and revised the manuscript for important intellectual content. JM designed the study, directly accessed and verified the data, conducted the statistical analysis, interpreted the results, and revised the manuscript for important intellectual content. MWD designed the study, directly accessed and verified the data, conducted the statistical analysis, interpreted the results, and drafted the manuscript.

All authors have accessed and verified the data, and were responsible for the decision to submit the manuscript.

## Data sharing statement

The dataset and related study documents will be made available to individual researchers upon reasonable request (*e.g.*, detailed research plan) to the corresponding author and following signing a data access agreement with the National Statistics Office of Mongolia.

## Declaration of interests

None of the authors has a financial or other conflict of interest in regards of data discussed in this manuscript.

## Appendix A Supplementary data

Summary translated
