# Supplementary material for: Prevalence, causes and outcomes of hospital admissions in Mongolia: a national registry-based descriptive analysis
Source: Lancet Reg Health West Pac. 2025 Jan 20;55:101470. doi: 10.1016/j.lanwpc.2025.101470 (PMC11831123; doi:10.1016/j.lanwpc.2025.101470)
Supplement: Supplementary file 1 [file mmc1.zip › Summary translated into Mongolian language _spl_1_spl_.odt]

ХУРААНГУЙ

Үндэслэл: Монгол улсын нийт эмнэлгүүдэд хэвтэн эмчлүүлсэн бүхий л тохиолдол, шалтгаан, үр дүн зэргийг агуулсан их өгөгдлийн сангийн судалгаа хийж нийтэлсэн цогц мэдээлэл, өнөөгийн байдлаар хараахан  үгүй байна.

Арга зүй: Судалгаанд 2019-01-р сараас 2023-12-р сарын хооронд эмнэлэгт хэвтэн эмчлүүлсэн Монгол улсын үндэсний эмнэлгийн мэдээллийн санд бүртгэгдсэн нийт насанд хүрэгсдийг хамруулсан их өгөгдлийн сангийн дүн шинжилгээ хийв. 

Тухайн судалгааны анхдагч болон хоёрдогч үр дүнгүүд нь эмнэлэгт хэвтэн эмчлүүлж буй тохиолдлын түвшин, зонхилох шалтгаан, эмнэлгийн ор хоног, эмнэлгийн нас баралтын түвшин зэргийг үндэсний  байв. Эмнэлэгт хэвтэн эмчлүүлэгчдийн их өгөгдлийн сангийн мэдээлэлд Хи-квадрат тест, Манн-Уитни-U-тест болон ANOVA Ранк тестийг ашиглан дүн шинжилгээ хийв. 

Үр дүн: Монгол улсын бүх шатлалын төрийн болон хувийн хэвшлийн нийт эмнэлгүүдэд хэвтэн эмчлүүлсэн 3,449,083 тохиолдлын бүртгэлийн санд дүн шинжилгээ хийсэн. Жил тутам 100,000 хүн амд ногдох эмнэлэгт хэвтэн эмчлүүлэгчдийн тохиолдлын дундаж 20,242 байна (IQR, 19,412-20,778). Судалгаанд хамруулсан он жилүүдэд эмнэлэгт хэвтэн эмчлүүлсэн зонхилох онош нь КОВИД-19 (8·7%), архаг нефрит, архаг пиелонефрит 6·7%; (алдаатай кодолсон байх үндэслэлтэй), артерийн гипертензи (2·5%), зүрхний архаг ишеми өвчин (2·3%), мэдрэлийн ёзоор, сүлжээний эмгэг (2·3%), даралт ихдэлтийн шалтгаант зүрхний эмгэг (2·1%), бүсэлхий нурууны өвдөлт (1·8%) зэрэг байгааг тодорхойлов. Эмнэлгийн дундаж ор хоног 7 (IQR, 6-9), эмнэлгийн нас баралтын түвшин 0.66% байна. Бүх үр дүнг эмэгтэй, эрэгтэй хүйс, КОВИД-19 цартахлын өмнөх, дэгдэлтийн болон дараах үе, оршин сууж буй бүс нутаг, эмнэлэгт хэвтсэн улирал, хэвтэн эмчлүүлж буй эмнэлгийн шатлал, улсын болон хувийн эмнэлгүүдээр нь харьцуулсан.

Дүгнэлт: Монгол Улсын нийт эмнэлгүүдэд 2019-2023 онуудад (КОВИД-19 цартахлын өмнөх, дэгдэлтийн, дараах үеүд) насанд хүрэгсдийн эмнэлэгт хэвтэх эмчлүүлэх тохиолдол үлэмж өндөр түвшинд байсныг тооцоолон тодорхойлов. Эмнэлэгт хэвтэн эмчлүүлэхэд хүргэж буй зонхилон тохиолдох оношийн бүлэгт хийсэн судалгаа шинжилгээний дүнгээр дээрх оношийн дагуу хэвтэн эмчлүүлэгчдийн ихэнх нь амбулаторийн болон анхан шатны тусламж үйлчилгээнд хамрагдан эмнэлэгт хэвтүүлэхгүйгээр эмчлүүлэх боломжтой эмгэг өвчин эзэлж байна. Эмнэлэгт хэвтэн эмчлүүлэх цочмог хийгээд хүндэвтэр, хүнд өвчин эмгэг харьцангуй цөөн хувийг эзэлдэг тул эмнэлгийн дундаж ор хоног зөвхөн 7 гэсэн үзүүлэлттэй байх, эмнэлгийн нөхцөл дэх нас баралт нэн бага түвшинд бүртгэгдэж байгаа зэрэгт нөлөөлж байна. 

Санхүүжилт: Байгууллагын санхүүжилттэй (Монголын Анагаахын Шинжлэх Ухааны Үндэсний Их Сургууль болон Иоханнес Кеплерийн Их Сургууль).

Түлхүүр үг: Монгол улс; эмнэлэгт хэвтэн эмчлүүлэх; тохиолдлын түвшин; эмнэлэгт хэвтсэн онош; эмнэлгийн ор хоног; нас баралт; КОВИД 19

This translation in Mongolian was submitted by the authors and we reproduce it as supplied. It has not been peer reviewed. Our editorial processes have only been applied to the original abstract in English, which should serve as reference for this manuscript.
