# Supplementary material for: Prevalence, causes and outcomes of hospital admissions in Mongolia: a national registry-based descriptive analysis
Source: Lancet Reg Health West Pac. 2025 Jan 20;55:101470. doi: 10.1016/j.lanwpc.2025.101470 (PMC11831123; doi:10.1016/j.lanwpc.2025.101470)
Supplement: Supplementary file 2 [file mmc2.zip › Electronic Supplementary Material.odt]

Electronic Supplementary Material to:

Prevalence, Causes and Outcomes of Hospital Admissions in Mongolia: A National Registry-based Descriptive Analysis

Altanchimeg Sainbayar, MD1,2; Prof. Ganbold Lundeg, MD1; Naranpurev Mendsaikhan, MD1,2; Prof. Jens Meier, MD3; Martin W. Dünser, MD3

1, Department of Critical Care and Anesthesia, Mongolian National University of Medical Sciences, Ulaanbaatar, Mongolia; 2, Intensive Care Unit, Mongolia Japan Hospital, Ulaanbaatar, Mongolia; 3, Department of Anesthesiology and Intensive Care Medicine, Kepler University Hospital and Johannes Kepler University, Linz, Austria

Table of Contents

Electronic Supplementary Material Table E1. ………………………………………………………………………………………………………………………………………………………………………….…3

Electronic Supplementary Material Table E2. ………………………………………………………………………………………………………………………………………………………………………….…5

Electronic Supplementary Material Table E3. ………………………………………………………………………………………………………………………………………………………………………….…9

Electronic Supplementary Material Table E4. ………………………………………………………………………………………………………………………………………………………………………….…13

Electronic Supplementary Material Table E5. ………………………………………………………………………………………………………………………………………………………………………….…15

Electronic Supplementary Material Table E6. ………………………………………………………………………………………………………………………………………………………………………….…17

Electronic Supplementary Material Figure E1. ....……………………………………………………………………………………………………………………………………………………………………….19

Electronic Supplementary Material Figure E2. ....……………………………………………………………………………………………………………………………………………………………………….20

Electronic Supplementary Material Figure E3. ....……………………………………………………………………………………………………………………………………………………………………….21

Electronic Supplementary Material Figure E4. ....……………………………………………………………………………………………………………………………………………………………………….22

Electronic Supplementary Material Figure E5. ....……………………………………………………………………………………………………………………………………………………………………….23

Electronic Supplementary Material Table E1. The twenty most common main diagnoses in patients categorized by the levels of admission hospitals.

| Level I |  | Level II |  | Level III |
| --- | --- | --- | --- | --- |
| n=572,301 | n=2,302,244 | n=574,538 |
| 1 | Chronic tubulo-interstitial nephritis | 79,320 (13·9%) | 1 | COVID-19 | 189,795 (8·2%) | 1 | COVID-19 | 34,432 (6%) |
| 2 | COVID-19 | 75,182 (13·1%) | 2 | Chronic tubulo-interstitial nephritis | 134,951 (5·9%) | 2 | Cholelithiasis | 18,191 (3·2%) |
| 3 | Chronic ischaemic heart disease | 26,695 (4·7%) | 3 | Chronic arterial hypertension | 60,283 (2·6%) | 3 | Liver and bile duct malignancy | 16,580 (2·9%) |
| 4 | Nerve roots/plexus disorders | 21,976 (3·8%) | 4 | Nerve root/plexus disorders | 50,848 (2·2%) | 4 | Chronic tubulo-interstitial nephritis | 15,999 (2·8%) |
| 5 | Hypertensive heart disease | 19,112 (3·3%) | 5 | Lower back pain | 42,347 (1·8%) | 5 | Chronic arterial hypertension | 11,270 (2%) |
| 6 | Lower respiratory tract infections | 16,378 (2·9%) | 6 | Chronic ischaemic heart disease | 41,520 (1·8%) | 6 | Complications of pregnancy termination | 10,672 (1·9%) |
| 7 | Polyosteoarthritis | 14,898 (2·6%) | 7 | Diabetes mellitus type II | 38,618 (1·7%) | 7 | Other cerebrovascular diseases | 9,645 (1·7%) |
| 8 | Chronic arterial hypertension | 14,533 (2·5%) | 8 | Other cerebrovascular diseases | 37,693 (1·6%) | 8 | Liver fibrosis or cirrhosis | 9,479 (1·6%) |
| 9 | Lower back pain | 12,462 (2·2%) | 9 | Liver fibrosis or cirrhosis | 33,919 (1·5%) | 9 | Diabetes mellitus type II | 8,885 (1·5%) |
| 10 | Sequelae of cerebrovascular disease | 10,129 (1·8%) | 10 | Cholelithiasis | 33,703 (1·5%) | 10 | Malignancy of the stomach | 8,183 (1·4%) |
| 11 | Other headache syndromes | 9,256 (1·6%) | 11 | False/premature labour | 31,548 (1·4%) | 11 | Acute appendicitis | 8,085 (1·4%) |
| 12 | Angina pectoris | 8,521 (1·5%) | 12 | Lower respiratory tract infections | 31,536 (1·4%) | 12 | Age-related cataract | 7,918 (1·4%) |
| 13 | Gastritis and duodenitis | 8,479 (1·5%) | 13 | Other headache syndromes | 30,265 (1·3%) | 13 | Other and unspecified disorders of the nose and nasal sinuses | 7,232 (1·3%) |
| 14 | Other diseases of the liver | 6,810 (1·2%) | 14 | Sequelae of cerebrovascular disease | 29,503 (1·3%) | 14 | Knee osteoarthritis | 6,809 (1·2%) |
| 15 | Chronic hepatitis | 6,586 (1·2%) | 15 | Alcohol-related disorders | 29,273 (1·3%) | 15 | Nerve root/plexus disorders | 6,597 (1·1%) |
| 16 | Other arthritis | 6,510 (1·1%) | 16 | Complications of pregnancy termination | 28,970 (1·3%) | 16 | Fractures of the lower leg including the ankle | 6,218 (1·1%) |
| 17 | Dystonia | 6,392 (1·1%) | 17 | Angina pectoris | 27,109 (1·2%) | 17 | Intracranial injury | 6,044 (1·1%) |
| 18 | Knee osteoarthritis | 6,281 (1·1%) | 18 | Acute appendicitis | 26,056 (1·1%) | 18 | Lower back pain | 5,787 (1%) |
| 19 | Other inflammatory liver diseases | 6,127 (1·1%) | 19 | Gastritis and duodenitis | 23,123 (1%) | 19 | Lower respiratory tract infections | 5,406 (0·9%) |
| 20 | Other diseases of the pancreas | 5,759 (1%) | 20 | Chronic hepatitis | 22,644 (1%) | 20 | Angina pectoris | 5,381 (0·9%) |

COVID-19, corona virus disease 2019.

Electronic Supplementary Material Table E2. The twenty most common main diagnoses in patients categorized by their region of residence.

| Centre |  | North |  | South |
| --- | --- | --- | --- | --- |
| n=290,702 | n=685,559 | n=244,894 |
| 1 | COVID-19 | 27,079 (9·3%) | 1 | Chronic tubulo-interstitial nephritis | 64,683 (9·4%) | 1 | COVID-19 | 28,349 (11·6%) |
| 2 | Chronic tubulo-interstitial nephritis | 22,866 (7·9%) | 2 | COVID-19 | 58,407 (8·5%) | 2 | Chronic tubulo-interstitial nephritis | 21,994 (9%) |
| 3 | Chronic ischaemic heart disease | 8,068 (2·8%) | 3 | Chronic ischaemic heart disease | 20,749 (3%) | 3 | Chronic ischaemic heart disease | 6,763 (2·8%) |
| 4 | Nerve root/plexus disorders | 7,556 (2·6%) | 4 | Hypertensive heart disease | 18,479 (2·7%) | 4 | Hypertensive heart disease | 5,976 (2·4%) |
| 5 | Hypertensive heart disease | 6,795 (2·3%) | 5 | Chronic arterial hypertension | 17,748 (2·6%) | 5 | Nerve root/plexus disorders | 5,447 (2·2%) |
| 6 | Lower respiratory tract infections | 5,679 (2%) | 6 | Angina pectoris | 15,296 (2·2%) | 6 | Lower respiratory tract infections | 5,364 (2·2%) |
| 7 | Chronic arterial hypertension | 5,036 (1·7%) | 7 | Nerve root/plexus disorders | 12,673 (1·8%) | 7 | Chronic arterial hypertension | 4,435 (1·8%) |
| 8 | Other cerebrovascular diseases | 4,620 (1·6%) | 8 | Lower back pain | 10,779 (1·6%) | 8 | Other cerebrovascular diseases | 4,410 (1·8%) |
| 9 | Lower back pain | 4,274 (1·5%) | 9 | Diabetes mellitus type II | 10,208 (1·5%) | 9 | Cholelithiasis | 3,322 (1·4%) |
| 10 | Sequelae of cerebrovascular disease | 4,258 (1·5%) | 10 | Cholelithiasis | 9,600 (1·4%) | 10 | Gastritis and duodenitis | 3,281 (1·3%) |
| 11 | Cholelithiasis | 4,220 (1·5%) | 11 | Other cerebrovascular diseases | 9,250 (1·3%) | 11 | Lower back pain | 3,018 (1·2%) |
| 12 | Liver fibrosis or cirrhosis | 3,479 (1·2%) | 12 | Lower respiratory tract infections | 9,240 (1·3%) | 12 | Other headache syndromes | 3,010 (1·2%) |
| 13 | Angina pectoris | 3,382 (1·2%) | 13 | Sequelae of cerebrovascular disease | 8,365 (1·2%) | 13 | Other diseases of the pancreas | 2,958 (1·2%) |
| 14 | Acute appendicitis | 3,330 (1·1%) | 14 | Liver fibrosis or cirrhosis | 8,003 (1·2%) | 14 | Liver and bile duct malignancy | 2,873 (1·2%) |
| 15 | Other headache syndromes | 3,325 (1·1%) | 15 | Other headache syndromes | 7,985 (1·2%) | 15 | Acute appendicitis | 2,796 (1·1%) |
| 16 | Diabetes mellitus type II | 3,170 (1·1%) | 16 | Liver and bile duct malignancy | 7,647 (1·1%) | 16 | Polyosteoarthritis | 2,695 (1·1%) |
| 17 | Liver and bile duct malignancy | 3,083 (1·1%) | 17 | Acute appendicitis | 7,203 (1·1%) | 17 | Angina pectoris | 2,646 (1·1%) |
| 18 | Skin abscess, furuncle and carbuncle | 3,018 (1%) | 18 | Other diseases of the pancreas | 6,258 (0·9%) | 18 | Sequelae of cerebrovascular disease | 2,383 (1%) |
| 19 | Complications of pregnancy termination | 2,970 (1%) | 19 | Gastritis and duodenitis | 5,773 (0·8%) | 19 | Liver fibrosis or cirrhosis | 2,381 (1%) |
| 20 | Polyosteoarthritis | 2,922 (1%) | 20 | Alcohol-related disorders | 5,586 (0·8%) | 20 | Skin abscess, furuncle and carbuncle | 2,311 (0·9%) |

| East |  | West |  | Ulaanbaatar |
| --- | --- | --- | --- | --- |
| n=239,201 | n=397,877 | n=1,468,491 |
| 1 | COVID-19 | 23,642 (9·9%) | 1 | Chronic tubulo-interstitial nephritis | 37,138 (9·3%) | 1 | COVID-19 | 128,656 (8·8%) |
| 2 | Chronic tubulo-interstitial nephritis | 14,012 (5·9%) | 2 | COVID-19 | 24,138 (6·1%) | 2 | Chronic tubulo-interstitial nephritis | 65,105 (4·4%) |
| 3 | Nerve root/plexus disorders | 7,710 (3·2%) | 3 | Chronic arterial hypertension | 13,074 (3·3%) | 3 | Chronic arterial hypertension | 40,078 (2·7%) |
| 4 | Lower back pain | 5,545 (2·3%) | 4 | Lower back pain | 11,308 (2·8%) | 4 | Nerve root/plexus disorders | 36,827 (2·5%) |
| 5 | Chronic ischaemic heart disease | 4,949 (2·1%) | 5 | Hypertensive heart disease | 10,885 (2·7%) | 5 | Complications of pregnancy termination | 27,701 (1·9%) |
| 6 | Chronic arterial hypertension | 4,684 (2%) | 6 | Other headache syndromes | 10,133 (2·5%) | 6 | Diabetes mellitus type II | 27,676 (1·9%) |
| 7 | Lower respiratory tract infections | 4,181 (1·7%) | 7 | Chronic ischaemic heart disease | 9,613 (2·4%) | 7 | Cholelithiasis | 27,368 (1·9%) |
| 8 | Hypertensive heart disease | 4,017 (1·7%) | 8 | Lower respiratory tract infections | 8,244 (2·1%) | 8 | Chronic ischaemic heart disease | 26,845 (1·8%) |
| 9 | Other headache syndromes | 3,903 (1·6%) | 9 | Nerve root/plexus disorders | 7,781 (2%) | 9 | Liver fibrosis or cirrhosis | 25,912 (1·8%) |
| 10 | Angina pectoris | 3,795 (1·6%) | 10 | Cholecystitis | 6,808 (1·7%) | 10 | Hypertensive heart disease | 24,825 (1·7%) |
| 11 | Liver and bile duct malignancy | 3,719 (1·6%) | 11 | Sequelae of cerebrovascular disease | 6,184 (1·6%) | 11 | Lower back pain | 22,791 (1·6%) |
| 12 | Cholelithiasis | 3,631 (1·5%) | 12 | False/premature labour | 5,723 (1·4%) | 12 | False/premature labour | 22,524 (1·5%) |
| 13 | Liver fibrosis or cirrhosis | 3,491 (1·5%) | 13 | Other cerebrovascular diseases | 5,612 (1·4%) | 13 | Other cerebrovascular diseases | 22,264 (1·5%) |
| 14 | Brucellosis | 3,424 (1·4%) | 14 | Acute appendicitis | 5,020 (1·3%) | 14 | Alcohol-related disorders | 18,551 (1·3%) |
| 15 | Other cerebrovascular diseases | 3,065 (1·3%) | 15 | Angina pectoris | 4,629 (1·2%) | 15 | Dystonia | 17,789 (1·2%) |
| 16 | Other arthritis | 2,926 (1·2%) | 16 | Cholelithiasis | 4,237 (1·1%) | 16 | Lower respiratory tract infections | 17,762 (1·2%) |
| 17 | Sequelae of cerebrovascular disease | 2,806 (1·2%) | 17 | Knee osteoarthritis | 4,077 (1%) | 17 | Sequelae of cerebrovascular disease | 17,205 (1·2%) |
| 18 | Knee osteoarthritis | 2,742 (1·1%) | 18 | Gastritis and duodenitis | 4,012 (1%) | 18 | Chronic hepatitis | 17,063 (1·1%) |
| 19 | Gastritis and duodenitis | 2,680 (1·1%) | 19 | Liver fibrosis and cirrhosis | 3,985 (1%) | 19 | Liver and bile duct malignancy | 15,610 (1·1%) |
| 20 | Polyosteoarthritis | 2,619 (1·1%) | 20 | Liver and bile duct malignancy | 3,717 (0·9%) | 20 | Gastritis and duodenitis | 15,030 (1%) |

COVID-19, corona virus disease 2019.

Electronic Supplementary Material Table E3. The twenty most common main diagnoses in patients categorized by the season of admission.

| Winter |  | Spring |  | Summer |
| --- | --- | --- | --- | --- |
| n=805,456 | n=886,535 | n=827,535 |
| 1 | Chronic tubulo-interstitial nephritis | 59,766 (7·4%) | 1 | Chronic tubulo-interstitial nephritis | 66,487 (7·5%) | 1 | COVID-19 | 93,722 (11·3%) |
| 2 | COVID-19 | 44,692 (5·5%) | 2 | COVID-19 | 27,029 (3%) | 2 | Chronic tubulo-interstitial nephritis | 51,675 (6·2%) |
| 3 | Chronic arterial hypertension | 22,336 (2·8%) | 3 | Chronic arterial hypertension | 26,988 (3%) | 3 | Nerve root/plexus disorders | 18,957 (2·3%) |
| 4 | Chronic ischaemic heart disease | 20,809 (2·6%) | 4 | Chronic ischaemic heart disease | 23,285 (2·6%) | 4 | Chronic arterial hypertension | 16,959 (2%) |
| 5 | Lower respiratory tract infections | 19,683 (2·4%) | 5 | Nerve root/plexus disorders | 22,057 (2·5%) | 5 | Hypertensive heart disease | 16,668 (2%) |
| 6 | Nerve root/plexus disorders | 19,208 (2·4%) | 6 | Hypertensive heart disease | 21,092 (2·4%) | 6 | Lower back pain | 14,289 (1·7%) |
| 7 | Hypertensive heart disease | 16,544 (2·1%) | 7 | Lower back pain | 16,513 (1·9%) | 7 | Cholelithiasis | 13,135 (1·6%) |
| 8 | Lower back pain | 14,849 (1·8%) | 8 | Diabetes mellitus type II | 15,008 (1·7%) | 8 | Other cerebrovascular diseases | 12,165 (1·5%) |
| 9 | Cholelithiasis | 12,543 (1·6%) | 9 | Cholelithiasis | 14,824 (1·7%) | 9 | Diabetes mellitus type II | 11,816 (1·4%) |
| 10 | Angina pectoris | 11,717 (1·5%) | 10 | Other cerebrovascular diseases | 14,776 (1·7%) | 10 | Liver fibrosis or cirrhosis | 11,340 (1·4%) |
| 11 | Other cerebrovascular diseases | 11,654 (1·4%) | 11 | Liver fibrosis or cirrhosis | 13,608 (1·5%) | 11 | Complications of pregnancy termination | 10,206 (1·2%) |
| 12 | Diabetes mellitus type II | 11,566 (1·4%) | 12 | Lower respiratory tract infections | 12,994 (1·5%) | 12 | Sequelae of cerebrovascular disease | 10,003 (1·2%) |
| 13 | Liver fibrosis or cirrhosis | 11,565 (1·4%) | 13 | Angina pectoris | 11,855 (1·3%) | 13 | Other headache syndromes | 9,789 (1·2%) |
| 14 | Other headache syndromes | 10,890 (1·4%) | 14 | Sequelae of cerebrovascular disease | 11,832 (1·3%) | 14 | False/premature labour | 9,710 (1·2%) |
| 15 | False/premature labour | 9,918 (1·2%) | 15 | Other headache syndromes | 11,822 (1·3%) | 15 | Acute appendicitis | 9,495 (1·1%) |
| 16 | Sequelae of cerebrovascular disease | 9,425 (1·2%) | 16 | Complications of pregnancy termination | 10,967 (1·2%) | 16 | Angina pectoris | 8,669 (1%) |
| 17 | Complications of pregnancy termination | 9,406 (1·2%) | 17 | False/premature labour | 10,467 (1·2%) | 17 | Liver and bile duct malignancy | 8,652 (1%) |
| 18 | Liver and bile duct malignancy | 8,731 (1·1%) | 18 | Liver and bile duct malignancy | 10,171 (1·1%) | 18 | Gastritis and duodenitis | 8,608 (1%) |
| 19 | Acute appendicitis | 8,697 (1·1%) | 19 | Acute appendicitis | 9,610 (1·1%) | 19 | Lower respiratory tract infections | 8,243 (1%) |
| 20 | Gastritis and duodenitis | 8,417 (1%) | 20 | Gastritis and duodenitis | 9,264 (1%) | 20 | Other diseases of the pancreas | 7,976 (1%) |

| Autumn |
| --- |
| n=929,557 |
| 1 | COVID-19 | 133,966 (14·4%) |
| 2 | Chronic tubulo-interstitial nephritis | 52,511 (5·6%) |
| 3 | Hypertensive heart disease | 19,293 (2·1%) |
| 4 | Nerve root/plexus disorders | 19,119 (2·1%) |
| 5 | Chronic ischaemic heart disease | 19,018 (2%) |
| 6 | Chronic arterial hypertension | 18,633 (2%) |
| 7 | Lower back pain | 14,963 (1·6%) |
| 8 | Cholelithiasis | 13,791 (1·5%) |
| 9 | Other cerebrovascular diseases | 13,349 (1·4%) |
| 10 | Diabetes mellitus type II | 13,269 (1·4%) |
| 11 | Liver fibrosis or cirrhosis | 12,562 (1·4%) |
| 12 | Lower respiratory tract infections | 12,412 (1·3%) |
| 13 | Sequelae of cerebrovascular disease | 11,396 (1·2%) |
| 14 | Other headache syndromes | 10,276 (1·1%) |
| 15 | False/premature labour | 10,134 (1·1%) |
| 16 | Complications of pregnancy termination | 9,986 (1·1%) |
| 17 | Liver and bile duct malignancy | 9,824 (1·1%) |
| 18 | Angina pectoris | 8,782 (0·9%) |
| 19 | Knee osteoarthritis | 8,558 (0·9%) |
| 20 | Alcohol-related disorders | 8,507 (0·9%) |

COVID-19, corona virus disease 2019.

Electronic Supplementary Material Table E4. The twenty most common main diagnoses in patients categorized by the type of the admission hospital.

| Public Hospitals |  | Private Hospitals |
| --- | --- | --- |
| n=2,563,091 | n=885,944 |
| 1 | COVID-19 | 271,348 (10·5%) | 1 | Chronic tubulo-interstitial nephritis | 72,025 (8·1%) |
| 2 | Chronic tubulo-interstitial nephritis | 158,414 (6·2%) | 2 | COVID-19 | 28,061 (3·2%) |
| 3 | Chronic ischaemic heart disease | 64,969 (2·5%) | 3 | Lower back pain | 25,693 (2·9%) |
| 4 | Chronic arterial hypertension | 64,459 (2·5%) | 4 | Chronic arterial hypertension | 21,754 (2·5%) |
| 5 | Nerve root/plexus disorders | 59,139 (2·3%) | 5 | Hypertensive heart disease | 20,800 (2·3%) |
| 6 | Hypertensive heart disease | 52,797 (1·2%) | 6 | Nerve root/plexus disorders | 20,202 (2·3%) |
| 7 | Lower respiratory tract infections | 47,540 (1·9%) | 7 | Chronic ischaemic heart disease | 15,102 (1·7%) |
| 8 | Cholelithiasis | 43,642 (1·7%) | 8 | Angina pectoris | 14,578 (1·6%) |
| 9 | Diabetes mellitus type II | 39,945 (1·6%) | 9 | Other cerebrovascular diseases | 13,417 (1·5%) |
| 10 | Liver fibrosis or cirrhosis | 38,560 (1·5%) | 10 | Knee osteoarthritis | 12,663 (1·4%) |
| 11 | Other cerebrovascular diseases | 38,527 (1·5%) | 11 | Other headache syndromes | 12,004 (1·4%) |
| 12 | False/premature labour | 38,509 (1·5%) | 12 | Gastritis and duodenitis | 11,900 (1·3%) |
| 13 | Complications of pregnancy termination | 34,938 (1·4%) | 13 | Diabetes mellitus type II | 11,714 (1·3%) |
| 14 | Lower back pain | 34,921 (1·4%) | 14 | Spondylosis | 11,139 (1·3%) |
| 15 | Sequelae of cerebrovascular disease | 33,484 (1·3%) | 15 | Dystonia | 10,778 (1·2%) |
| 16 | Liver and bile duct malignancy | 31,249 (1·2%) | 16 | Chronic hepatitis | 10,757 (1·2%) |
| 17 | Acute appendicitis | 31,143 (1·2%) | 17 | Cholelithiasis | 10,651 (1·2%) |
| 18 | Other headache syndromes | 30,773 (1·2%) | 18 | Liver fibrosis or cirrhosis | 10,515 (1·2%) |
| 19 | Angina pectoris | 26,445 (1%) | 19 | Age-related cataract | 10,374 (1·2%) |
| 20 | Alcohol-related disorders | 25,442 (1%) | 20 | Other brain disorders | 10,211 (1·2%) |

COVID-19, corona virus disease 2019.

Electronic Supplementary Material Table E5. Length of hospital stay in the study population and defined subgroups.

|  | Length of Stay | p-value |
| --- | --- | --- |
| Sex |  | <0·001* |
| females | 7 (6-8) |  |
| males | 7 (6-9) |  |
| Region of residencea |  | <0·001* |
| East | 7 (7-9) |  |
| North | 7 (6-9) |  |
| Centre | 7 (6-9) |  |
| South | 7 (6-9) |  |
| West | 7 (7-9) |  |
| Ulaanbaatar | 7 (5-8) |  |
| Season of admission |  | <0·001* |
| winter | 7 (6-9) |  |
| spring | 7 (6-9) |  |
| summer | 7 (6-9) |  |
| autumn | 7 (6-8) |  |
| COVID-19 pandemic |  | <0·001* |
| before (2019-2020) | 7 (6-9) |  |
| during (2021-2022) | 7 (6-9) |  |
| after (2023) | 7 (6-7) |  |
| Level of admission hospital |  | <0·001* |
| level I | 7 (7-8) |  |
| level II | 7 (6-9) |  |
| level III | 7 (4-8) |  |
| Type of admission hospital |  | <0·001* |
| public | 7 (6-9) |  |
| private | 7 (5-8) |  |

COVID-19, corona virus disease 2019.

*, significant difference between subgroups (Mann-Whitney U-rank sum test or ANOVA on ranks).

a, regions include the following aimags: East, Dornod, Sukhbataar and Khentii, North: Arkhangai, Bulgan, Orkhon, Khuvsgul, Selenge and Darkhna-Uul, Centre: Dornogobi, Tuv and Uvurkhangai, South: Bayankhongor, Umnugobi, Gobisumber and Dundgobi, West: Bayan-Ulgii, Gobi-Altai, Zavkhan, Uvs and Khovd, Ulaanbaatar: capital city of Ulaanbaatar.

Electronic Supplementary Material Table E6. All-cause crude hospital mortality rates in defined subgroups.

|  | Survivors | Non-Survivors | Mortality | p-value |
| --- | --- | --- | --- | --- |
|  | n | n | % |  |
| Sex |  |  |  | <0·001* |
| females | 2,162,223 | 9,137 | 0·42% |  |
| males | 1,264,177 | 13,546 | 1·1% |  |
| Region of residencea |  |  |  | <0·001* |
| East | 237,563 | 1,638 | 0·68% |  |
| North | 681,590 | 3,969 | 0·58% |  |
| Centre | 288,649 | 2,053 | 0·71% |  |
| South | 243,466 | 1,428 | 0·58% |  |
| West | 396,480 | 1,397 | 0·35% |  |
| Ulaanbaatar | 1,456,657 | 11,834 | 0·81% |  |
| Season of admission |  |  |  | <0·001* |
| winter | 800,260 | 5,196 | 0·65% |  |
| spring | 881,298 | 5,237 | 0·59% |  |
| summer | 821,773 | 5,762 | 0·7% |  |
| autumn | 923,069 | 6,488 | 0·7% |  |
| COVID-19 pandemic |  |  |  | <0·001* |
| before (2019-2020) | 1,256,118 | 7,453 | 0·59% |  |
| during (2021-2022) | 1,424,698 | 10,517 | 0·73% |  |
| after (2023) | 745,584 | 4,713 | 0·63% |  |
| Level of admission hospital |  |  |  | <0·001* |
| level I | 568,454 | 2,313 | 0·41% |  |
| level II | 2,290,857 | 11,387 | 0·49% |  |
| level III | 565,562 | 8,976 | 1·56% |  |
| Type of admission hospital |  |  |  | <0·001* |
| public | 2,542,036 | 21,055 | 0·61% |  |
| private | 884,316 | 1,628 | 0·05% |  |

COVID-19, corona virus disease 2019.

*, significant difference between subgroups (Chi-square test).

a, regions include the following aimags: East, Dornod, Sukhbataar and Khentii, North: Arkhangai, Bulgan, Orkhon, Khuvsgul, Selenge and Darkhna-Uul, Centre: Dornogobi, Tuv and Uvurkhangai, South: Bayankhongor, Umnugobi, Gobisumber and Dundgobi, West: Bayan-Ulgii, Gobi-Altai, Zavkhan, Uvs and Khovd, Ulaanbaatar: capital city of Ulaanbaatar.

Electronic Supplementary Material Figure E1. Map of Mongolia indicating the six regions of residence as applied in this study.

Electronic Supplementary Material Figure E2. Study flow diagram.

Electronic Supplementary Material E3. Missingness map.

Electronic Supplementary Material Figure E4. Absolute number of hospital admissions and age distribution among male and female study patients.

Electronic Supplementary Material Figure E5. Histogram of the length of hospital stays in the study population.

For illustrative reasons the length of hospital stay in days was truncated at day 14. Two percent of cases included in this analysis had a hospital length of stay >14 days.
